# Role of IGF-1 pathway in lung fibroblast activation

**DOI:** 10.1186/1465-9921-14-102

**Published:** 2013-10-08

**Authors:** Chi F Hung, Maryam G Rohani, Sung-soon Lee, Peter Chen, Lynn M Schnapp

**Affiliations:** 1 Center for Lung Biology, Division of Pulmonary and Critical Care Medicine, Department of Medicine, University of Washington, 850 Republican St, Box358052, Seattle 98109, WA, USA

**Keywords:** Fibroblasts, IGF, Fibrosis

## Abstract

**Background:**

IGF-1 is elevated in pulmonary fibrosis and acute lung injury, where fibroblast activation is a prominent feature. We previously demonstrated that blockade of IGF pathway in murine model of lung fibrosis improved outcome and decreased fibrosis. We now expand that study to examine effects of IGF pathway on lung fibroblast behaviors that could contribute to fibrosis.

**Methods:**

We first examined mice that express αSMA promoter upstream of GFP reporter treated with A12, a blocking antibody to IGF-1 receptor, after bleomycin induced lung injury. We then examined the effect of IGF-1 alone, or in combination with the pro-fibrotic cytokine TGFβ on expression of markers of myofibroblast activation *in vitro*, including αSMA, collagen α1, type 1, collagen α1, type III, and TGFβ expression.

**Results:**

After bleomycin injury, we found decreased number of αSMA-GFP + cells in A12 treated mice, validated by αSMA immunofluorescent staining. We found that IGF-1, alone or in combination with TGF-β, did not affect αSMA RNA expression, promoter activity, or protein levels when fibroblasts were cultured on stiff substrate. IGF-1 stimulated *Col1a1* and *Col3a1* expression on stiff substrate. In contrast, IGF-1 treatment on soft substrate resulted in upregulation of αSMA gene and protein expression, as well as *Col1a1* and *Col3a1* transcripts. In conclusion, IGF-1 stimulates differentiation of fibroblasts into a myofibroblast phenotype in a soft matrix environment and has a modest effect on αSMA stress fiber organization in mouse lung fibroblasts.

## Introduction

Insulin-like growth factor-1 (IGF-1) plays an important role in the development and homeostasis of many organs. IGF-1 acts as an important survival factor for various cells by inhibiting apoptosis and inducing cellular proliferation [1-3]. However, IGF-1 has also been implicated in disease states where pathologic fibrosis is the predominant feature. For example, in patients with systemic sclerosis (SSc), serum IGF-1 level is elevated in those with more severe skin involvement and pulmonary fibrosis [4]. Moreover, affected skin from subjects with SSc show 1.9 fold higher levels of IGF-1 mRNA expression compared to normal controls [4]. In the murine bleomycin lung injury model, IGF-1 mRNA was increased three to four fold over control in pulmonary fibrosis [5]. IGF-1 immunostaining was increased in lung tissues from patients with fibroproliferative ARDS [6] and IGF-1 levels were elevated in the broncho-alveolar lavage fluid (BALF) of patients with early ARDS [7]. We showed that IGF-1 provided a pro-survival signal to lung fibroblasts but not epithelial cells [8]. We further showed that blockade of the IGF-1 pathway in the murine bleomycin lung injury model hastened resolution of pulmonary fibrosis and increased fibroblast apoptosis [8]. In this study, we ask whether IGF-1 activates the myofibroblast phenotype. In addition to its role in cell survival, IGF-1 can alter gene expression and lead to phenotypic changes in fibroblasts [9-12]. The myofibroblast phenotype confers a number of important functional changes that play an important role in lung injury and repair [13,14]. We hypothesize that the IGF-1 pathway increases fibrosis in lung injury by activating fibroblasts to the αSMA-expressing myofibroblast phenotype.

## Materials and methods

### Cells and reagents

Recombinant IGF-1 and TGF-β1 were purchased from R&D Systems (Minneapolis, MN). Function-blocking antibody to the human IGF-1 receptor (A12) and keyhole limpet hemocyanin (KLH) isotype control antibody were a generous gift from Dale Ludwig (ImClone Systems) [15,16]. A12 inhibits type 1 IGF receptor signaling in murine and human tissues and does not cross-react with the insulin receptor [15]. We verified that our preparation of A12 was endotoxin-free by Limulus Amebocyte Lysate assay (Cambrex BioScience). For detection of αSMA by Western blot, antibody to αSMA (mouse IgG) was purchased from Sigma-Aldrich (clone 1A4). Horseradish peroxidase-conjugated anti-mouse IgG were purchased from Zymed (San Francisco, CA).

### Bleomycin-induced lung injury

Animal protocol was approved by University of Washington Institutional Animal Care and Use Committee. Transgenic mice that express αSMA promoter upstream of GFP reporter construct on a C57Bl6 background (αSMA-GFP mice) were a generous gift from Dr. Jen-Yue Tsai (National Eye Institute, NIH) [17]. Mice underwent intratracheal bleomycin instillation (0.032U/mouse, SICOR Pharmaceuticals, Inc., Irvine, CA) as previously described [8]. Mice (n = 4/group) received injections of either A12 (40 mg/kg) or KLH isotype control antibody intraperitoneally on d7 following bleomycin instillation and then twice weekly. The mice were sacrificed 21 days after bleomycin instillation. The right lungs were inflated to 25 cm H2O pressure, fixed with paraformaldehyde and paraffin embedded. 5 μm thick sections were deparaffinized, rehydrated. For quantification of GFP (+) cells, right middle lobe sections were systematically scanned in a microscope using 10× objective. Total number of GFP positive cells and DAPI positive cells were quantified in each successive field (NIH ImageJ, v1.41o). The mean score of all the fields was used for each mouse. For quantification of αSMA (+) cells, rehydrated right lung sections underwent heat antigen retrieval in buffer (Dako Target Retrieval Solution), incubated in blocking solution overnight at 4C, and then immunostained with Cy3-conjugated anti-αSMA antibody (Sigma-Aldrich clone 1A4, 1:200) for 1 hr at room temperature. The sections were counterstained with DAPI and mounted for visualization (Invitrogen Prolong Gold). Five predetermined fields were examined on each slide with 10× objective. The degree of αSMA staining was expressed as the ratio of red Cy3 staining area to DAPI staining area (NIH ImageJ, v1.41o). Airway and vascular-associated αSMA were masked in the image analysis. For confocal microscopy, bleomycin-injured mouse lungs from αSMA-GFP mice were inflated with 4% paraformaldehyde and fixed for 2 hours, submerged in 18% sucrose at 4C overnight, and embedded and frozen in OCT. Frozen sections were immunostained for αSMA (Cy3-conjugated anti-αSMA, Sigma-Aldrich clone 1A4, 1:200) and visualized under confocal microscopy.

### *In vitro* IGF-1 studies

Mouse lung fibroblasts (MLF) isolated from C57/Bl6 or αSMA-GFP mice were maintained in DMEM with 10% FBS, 100 U/ml penicillin, 100 U/ml streptomycin and 5 mM glutamate at 37°C in 5% CO_2_ as previously described [18]. For some studies, MLF were isolated from C57/Bl6 mice three days after intratracheal instillation with saline (control) or bleomycin (n = 3 mice/group). Unless otherwise indicated, experiments used MLF from C57/Bl6 wildtype mice. Cells were used between passages 2-5. MLF were grown to subconfluence and then plated either in 6-well tissue culture plates (Falcon) or 6-well tissue culture plates coated with collagen matrix (1 mg/ml). To test the effect of a soft extracellular matrix on fibroblast response to IGF-1, we employed a collagen I gel matrix at a final concentration of 1 mg/ml, which has been previously described to have an elastic modulus of < 100 Pa [19]. We mixed Collagen I (3 mg/ml) (BD Biosciences), MCDB (2X), and DMEM (with or without resuspended MLF) in 1:1:1 ratio. Immediately following mixing, the pH of the mixture was adjusted to neutral using 1 M NaOH. The mixture was allowed to gelatinize at room temperature for 1 hour.

Following attachment, cells were serum-starved overnight and treated with IGF-1 (100 ng/ml), TGF-β1 (10 ng/ml or 1 ng/ml), or IGF-1 (100 ng/ml)/TGF-β1 (10 ng/ml) for 24 hr, with the presence of A12 (40 μg/ml) or PI3 kinase inhibitor LY294002 (Calbiochem, 50 μM) in some experiments. Controls were serum-free media alone, and with A12 or LY294002 in experiments where the inhibitors were used. Parallel cultures were used for immunofluorescence studies, protein analysis, RNA analysis and promoter activity. All experiments were performed in triplicate, and repeated at least 3 times.

### Real-time PCR

Total RNA was isolated from MLF using Qiagen RNeasy Mini Kit per manufacturer’s specifications after treatment with the indicated growth factors. RNA quality was verified using Agilent Bioanalyzer. Total RNA was reverse-transcribed to cDNA using Applied Biosystems High-Capacity cDNA Archive Kit. Real-time PCR was done using ABI7900HT with the use of pre-designed primer and probes (ABI TaqMan Gene Expression Assays) for *Hprt* (Mm00446968_m1), and *Acta2* (Mm01546133_m1), *Col1a1* (Mm00801666_g1), *Col3a1* (Mm01254476_m1), and *Tgfb1* (Mm00441724_m1). Analysis was done using MS Excel calculating RQ by 2-DDCT.

### αSMA promoter activity

MLF isolated from αSMA-GFP mice were washed with PBS, trypsinized and fixed in paraformaldehyde. Flow cytometry (3000 cells per treatment group) was performed using the Guava PCA System (Guava Technologies, Hayward, CA) with the Guava ExpressPlus program and data analyzed using CellQuest 2.0 (BD Biosciences).

### Western blot analysis

To assess αSMA protein expression, cells were washed in PBS and lysed in buffer containing 100 mM Tri-HCl (pH 7.4), 150 mM NaCl, 1 mM CaCl_2_, 0.1% SDS, 1% Triton-X, 0.1% NP-40, and protease inhibitor cocktail tablet (Roche). Protein concentrations were determined by the BCA assay (Pierce). Equal amounts of protein were separated by sodium dodecyl sulfate-polyacrylamide gel electrophoresis (SDS-PAGE), and electrophoretically transferred to PVDF membrane. Membranes were blocked with 5% nonfat dry milk/0.05% Tween-20/PBS for 1 hr at room temperature, incubated with mouse anti-αSMA IgG (1:20,000), rabbit anti-Collagen I IgG (GeneTex, 1:5,000), or rabbit anti-Collagen III IgG (Rockland, 1:5,000) overnight at 4°C, washed with 0.1% Tween-20/PBS, incubated with horseradish peroxidase-conjugated goat anti-mouse IgG (1:10,000) for 1 hr, washed with 0.1% Tween-20/PBS and then developed with enhanced chemiluminescence (ECL) technique (Amersham, England). Densitometric analysis of relative band intensities was performed by analyzing scanned blots with NIH Image J (version 1.41o). Values are normalized to GAPDH control and presented as relative intensities compared to control (serum-free condition).

### αSMA and filamentous actin (F-actin) Co-staining

To assess αSMA fiber organization, primary MLF at P1 were treated with IGF-1 (100 ng/ml), TGF-β1 (10 ng/ml) or IGF-1/TGF-β1 (100 ng/ml and 10 ng/ml, respectively) for 24 hr, then fixed with 4% paraformaldehyde at RT ×10 min followed by permeabilization with 0.5% Triton-X100 in PBS at RT × 3 min. The fixed cells were blocked in 1% BSA in PBS × 20 min at RT and incubated with primary antibody to αSMA (Abcam, rabbit IgG) overnight at 4°C, then incubated with Alexa488-conjugated secondary antibody (Invitrogen, goat anti-rabbit IgG), followed by incubation with Alexa 564-conjugated phalloidin (5 units/ml, Invitrogen) at RT for 20 min for F-actin staining. Nuclei were counterstained with DAPI. Ten to twelve random fields (20×) per treatment condition were analyzed for cells staining for αSMA stress fibers (green) and F-actin fibers (red). The total number of F-actin staining cells per field was counted. Of the F-actin + cells counted, the number of αSMA stress fiber + cells were counted. Results are presented as percentage of αSMA stress fiber + cells out of the total number of F-actin + cells. Imaging was performed with the assistance of the Lynn and Mike Garvey Cell Imaging Laboratory at the UW Institute for Stem Cell and Regenerative Medicine. Images were obtained using a Nikon TiE inverted widefield fluorescence microscope and analyzed by NIH ImageJ (version 1.41o).

### Statistical analysis

Means of more than two groups of data were compared using one-way analysis of variance (ANOVA) for analysis of one independent variable or two way ANOVA, for analysis of two independent variables, followed by Tukey’s honestly significant difference (HSD) post hoc test. Student T-test was used for comparison of parametric data. All tests were two-tailed and *p* values ≤ 0.05 were considered significant. Statistical analysis was performed using GraphPad Prism for Macintosh version 4.0c (GraphPad Software).

## Results

### Blockade of IGF-1 pathway *in vivo* decreases αSMA expression after injury

**Figure 1:**
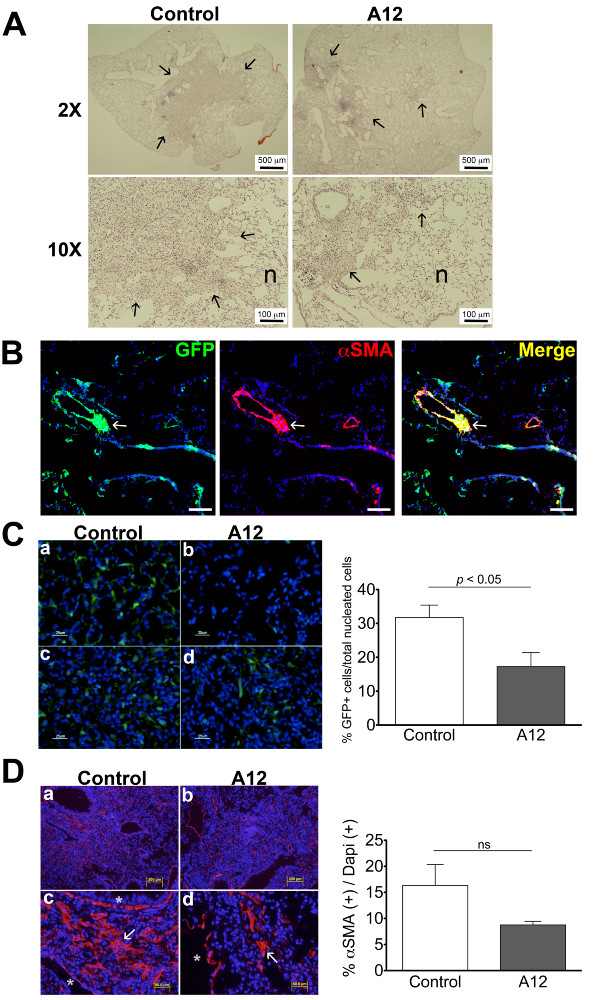
**Decreased αSMA promoter activity and αSMA protein expression in bleomycin-injured mice treated with IGF-1 receptor blocking antibody (A12). (A)** Representative H&E sections of αSMA-GFP mice at day 21 after bleomycin injury. Fibrotic regions of lung parenchyma are indicated by () and normal lung parenchyma are indicated by (**n**). **(B)** αSMA immunostaining of bleomycin-injured lung in an αSMA-GFP mouse. Note the overlap of αSMA staining (red) with αSMA-GFP expression (green) in the peribronchiolar fibrotic region indicated by (). Scale bars represent 100 μm. **(C)** (Left) Representative fluorescent images of αSMA-GFP mice treated with A12 **(b** and **d)** showed less αSMA promoter activity as indicated by GFP (green) positive cells, compared to control mice **(a** and **c)** at d21 after bleomycin instillation. (Right) Percentage of αSMA-GFP + cells/total number of DAPI + cells, quantification by NIH ImageJ (n = 4 mice/group, mean ± SEM). **(D)** (Left) Representative images of αSMA staining by immunofluorescent microscopy of the same A12-treated mice **(b** and **d)** compared to control mice **(a** and **c)** at d21 after bleomycin instillation. Large airways and vasculature staining for αSMA, indicated by an asterisk (*), were masked in the analysis. Interstitial staining, indicated by an arrow (), was included in the analysis. (Right) Ratio of αSMA staining area per DAPI + area (n = 4 mice/group, mean ± SEM).

We previously demonstrated that IGF-1 is upregulated in patients with acute lung injury and in mice following bleomycin-induced lung injury [7,8]. Furthermore, IGF-1 receptor blockade hastened resolution of fibrosis in mouse model of injury [8]. To determine whether IGF-1 blockade attenuates myofibroblast activation, we examined αSMA-GFP transgenic mice after bleomycin-induced lung injury with or without A12 antibody (IGF-1R antibody) treatment. Representative H&E images from bleomycin injured αSMA-GFP transgenic mice show decreased fibrotic regions in the peribronchiolar regions following bleomycin injury (Figure 1A). αSMA immunostaining shows co-localization of αSMA with GFP expression, demonstrating GFP expression as a reliable surrogate for αSMA expression following bleomycin injury in this transgenic model (Figure 1B). Following bleomycin-induced lung injury, we found significantly fewer GFP positive cells (as a marker of αSMA expression) (Figure 1C) in the A12 treated group. Interestingly, the most striking difference visually between the two groups was in areas of relatively normal architecture (a, b). To validate our findings in GFP, we immunostained lung sections for αSMA. We also found decreased αSMA expression in bleomycin-injured lungs that were treated with A12, consistent with our GFP findings (Figure 1D). In uninjured mice treated with A12 alone, there was no difference in GFP expression (not shown).

### Effect of IGF-1 treatment on αSMA promoter activity

In addition to an effect on cell survival, another potential explanation for decreased GFP (αSMA+) cells is a direct effect of IGF-1 blockade on fibroblast αSMA expression. Therefore, we asked whether IGF-1 affected fibroblast αSMA expression alone or synergistically with pro-fibrotic cytokine TGF-β1, and whether this effect can be blocked by treatment with the IGF-1 receptor-blocking antibody A12.

**Figure 2:**
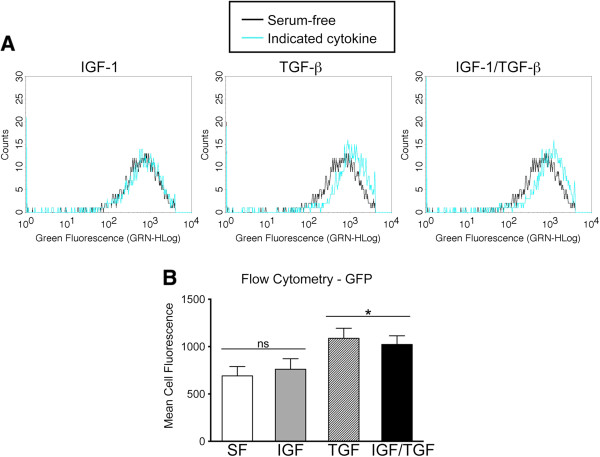
**Effect of IGF-1 on αSMA promoter activity in αSMA-GFP transgenic mice. (A)** MLF from αSMA-GFP mice were treated with IGF-1 (100 ng/ml), TGFβ (10 ng/ml), or both on tissue culture plate for 24 hr. Control group was maintained in serum-free media. Representative histogram of each treatment condition overlaying control group histogram is shown. **(B)** Bar graph summarizing the mean green fluorescence of each treatment condition. 3000 cells/condition analyzed. *p < 0.05 compared to serum-free control.

First, we evaluated αSMA promoter activity (measured by mean GFP intensity) in MLF isolated from αSMA-GFP mice. TGF-β1 but not IGF-1 increased αSMA promoter activity, and no synergistic effect was seen with IGF-1 and TGF-β1 co-stimulation (Figure 2). These results suggest IGF-1 does not regulate αSMA promoter activity in MLF either alone, or synergistically with TGF-β1, a cytokine known to upregulate αSMA RNA expression in the conditions tested.

### Effect of IGF-1 treatment on transcription of myofibroblast markers

**Figure 3:**
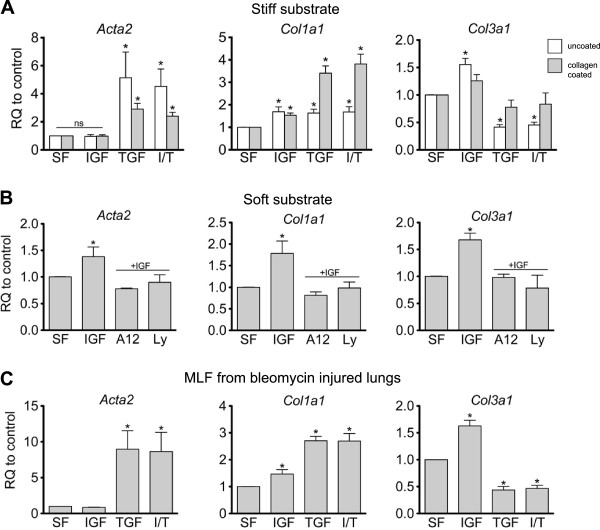
**Effect of matrix stiffness on response to IGF-1 treatment. (A)** MLF on tissue culture plate or collagen I-coated tissue culture plate (stiff substrates) were treated with IGF-1 (100 ng/ml), TGFβ (1 ng/ml) or IGF/TGFβ (100 ng/ml and 1 ng/ml, respectively), or serum-free media (negative control) for 24 hr. **(B)** MLF on collagen I (1 mg/ml) hydrogel (soft substrate) were treated with IGF-1 (100 ng/ml), IGF-1 (100 ng/ml) with A12 (40 μg/ml) or PI3 kinase inhibitor LY294002 (Ly, 50 μM;) for 24 h. **(C)** MLF isolated from bleomycin-injured C57Bl6 mice were treated with the indicated cytokine. Real time PCR analyses of myofibroblast markers *Acta2*, *Col1a1*, and *Col3a1* were performed. Data were normalized to HPRT expression. Y-axis represents fold increase compared to serum-free control (n = 3, mean ± SEM, *p < 0.05 compared to serum-free control).

Next, we assessed the role of IGF-1 on αSMA mRNA expression by real-time PCR. MLF grown on tissue-culture plate or collagen I-coated tissue culture plates (stiff substrates) were treated with IGF-1, TGF-β1 or combination of IGF-1 and TGF-β1 to assess for synergy. As expected, treatment with positive control TGF-β1 increased *Acta2* expression (Figure 3A). However, IGF-1 did not increase *Acta2* expression. Co-incubation of IGF-1 and TGF-β1 did not alter the TGF-β1 induced *Acta2* expression. Finally, treatment of cells with A12 did not change *Acta2* expression in any of the conditions, including TGF-β1 treatment (Additional file 1: Figure S1). We further confirmed that MLF isolated from bleomycin-injured mice did not respond differently to cytokine stimulation than MLF from uninjured mice: MLF isolated from bleomycin-injured mice and treated with IGF-1 did not increase αSMA RNA expression (Figure 3C).

αSMA expression is one of several changes observed with fibroblast activation. Activated fibroblasts may also increase TGF-β1 expression and synthesis of extracellular matrix proteins such as collagen α1, type I and collagen α1, type III. Therefore, we measured transcriptional changes in these genes after treatment with IGF-1, TGF-β1, or both. As expected, treatment with TGF-β1 increased *Col1a1* expression. Interestingly, IGF-1 treatment led to a 1.5 fold increase in *Col1a1* expression over serum-free control at 24 hours (Figure 3A) and the effect was inhibited by A12 (Additional file 1: Figure S1). IGF-1 treatment also led to a 1.5 fold increase in *Col3a1* expression.

The effect of IGF-1 treatment on MLF is not mediated through interaction with TGF-β1. No significant synergy was seen between IGF-1 and TGF-β1, and IGF-1 blockade with A12 did not affect TGF-β1 stimulated MLF (Additional file 1: Figure S1). Moreover, IGF-1 treatment no effect on *Tgfb1* expression in MLF (Additional file 2: Figure S2). Similarly, bleomycin did not affect MLF responsiveness to IGF-1 stimulation *in vitro* as similar results were obtained using MLF isolated from bleomycin-injured lungs (Figure 3C).

### Effect of IGF-1 treatment on αSMA and matrix protein expression

**Figure 4:**
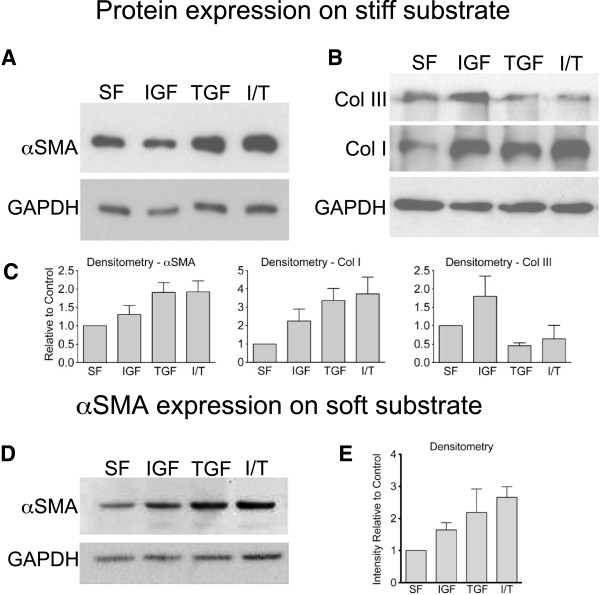
**Effect of IGF-1 on protein expression in soft and stiff substrates. (A**-**B)** MLF on tissue culture plate (stiff substrate) were stimulated with IGF-1, TGF-β1, or IGF-1/TGF-β1 for 24 hr. Representative Western blots of αSMA, Col I, and Col III with GAPDH loading control are shown. **(C)** Densitometry after normalization to loading control. **(D)** MLF cultured on soft matrix and treated with IGF, TGF-β1, or IGF/ TGF-β1 for 24 hr. Representative Western blots of αSMA with GAPDH loading control are shown. **(E)** Densitometry after normalization to loading control (n = 3, mean ± SEM).

Similar to findings with RNA expression, IGF-1 treatment on stiff substrate did not increase αSMA protein expression in MLFs after 24 hr (Figure 4A and C). TGF-β1 treatment on stiff substrate increased αSMA protein expression, but the addition of IGF-1 had no synergistic effect. Similarly, MLF isolated from bleomycin-injured mice did not show increased αSMA protein expression with IGF-1 treatment compared to MLF isolated from control mice (data not shown). In addition to αSMA, we also assessed *in vitro* expression of matrix proteins Col I and III. Consistent with our findings in *Col1a1* and *Col3a1* transcriptional activity, MLF treated with IGF-1 demonstrated increased Col I and III expression (Figure 4B and C).

### Effect of IGF-1 on stress fiber formation

**Figure 5:**
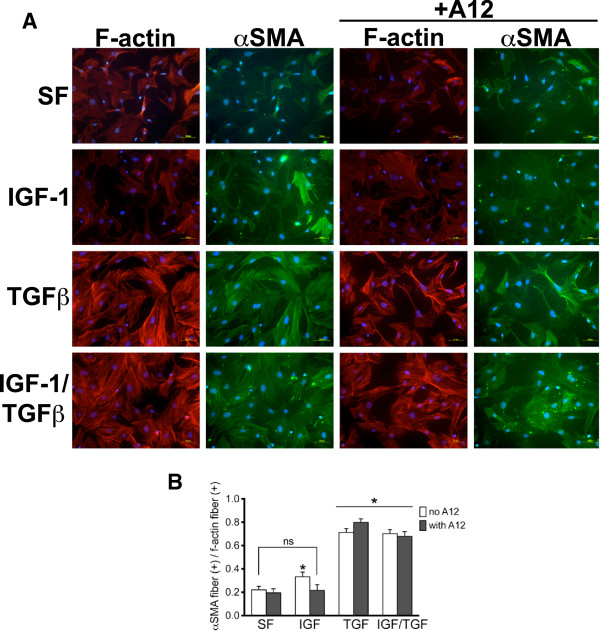
**IGF-1 treatment increases αSMA stress fibers. A**. MLF treated with IGF-1 (100 ng/ml), TGF-β1 (10 ng/ml) or IGF-1/TGF-β1 (100 ng/ml and 10 ng/ml, respectively) with or without A12 (40 μg/ml) for 24 hr. Negative control is serum free media. Cells were co-stained for F-actin (red) and αSMA (green). **B**. The mean ratio (±SEM) of αSMA stress fiber (+) fibroblasts over all F-actin (+) fibroblasts is presented in the bar graph. * *p* < 0.05 compared to serum-free control. ** *p* < <0.01 compared to serum-free control.

The percentage of cells staining for F-actin fibers (as indicated by phalloidin staining) and the intensity of F-actin fiber staining did not significantly change with IGF-1 treatment. However, IGF-1 treatment modestly increased the percentage of cells with αSMA-containing stress fibers compared to serum-free control (33% vs 19.5%, p = 0.05) (Figure 5). Furthermore, this increase was blocked by A12. As expected, TGF-β1 treatment increased the percentage of αSMA stress fiber positive fibroblasts and the intensity of phalloidin staining. However, there was no further increase in the percentage of fibroblasts with αSMA-containing stress fibers with the addition of IGF-1 to TGFβ treatment. A12 had no effect on TGF-β1-mediated increase in αSMA-containing stress fibers (Figure 5).

### Role of matrix stiffness in response to IGF stimulation

Since MLF rapidly differentiate into myofibroblasts when grown on tissue culture plastic, we questioned whether the high level of baseline myofibroblast activation obscured the effects of IGF-1 on αSMA expression. Therefore, we cultured MLF on collagen I gel matrix (soft substrate) and asked whether IGF-1 increased myofibroblast activation in these conditions. In contrast to MLF grown on tissue culture plastic, MLF grown on soft substrate up-regulated *Acta2* expression in response to IGF-1 (Figure 3B). Likewise, MLF grown on soft substrate significantly increased αSMA protein expression in response to IGF-1 (Figure 4D,E). IGF-1 treatment also increased *Col1a1* and *Col3a1* expression on soft substrate (Figure 3B). Treatment with TGF-β1, alone or in combination with IGF-1, resulted in a 2-fold increase in αSMA protein expression without synergistic effect with IGF-1 (Figure 4D,E). To ensure the soft biomechanical property of the collagen I gel substrate, rather than the presence of Collagen I in the substrate, was responsible for the effect of IGF-1 on αSMA, Col I and III, we also assessed the effect of IGF-1 on MLF cultured on collagen I-coated tissue culture plates. Similar to our findings in uncoated tissue culture plates, IGF-1 treatment had no effect on *Acta2* expression and stimulated *Col1a1* and *Col3a1* transcriptional activity (Figure 3A). Furthermore, the effect of IGF-1 on soft matrix was blocked by treatment with A12 blocking antibody (Figure 3B). We previously examined the signal transduction pathway activated by IGF-1 in MLF, and found that IGF-1 treatment led to phosphorylation of IRS-2 but not IRS-1, and phosphorylation of Akt [8]. The PI3-kinase pathwayis thus the likely pathway involved in IGF-1 signaling. When MLF grown on soft substrate was treated with PI3-kinase inhibitor Ly294002, the effect of IGF-1 on MLF was also blocked (Figure 3B). Together, these data suggest that the mechanical properties of the matrix modulate the response of MLF to IGF-1 stimulation.

## Discussion

IGF-1 has been reported to activate fibroblasts into the myofibroblast phenotype [9,12,20-22]. We previously demonstrated that IGF-1 provided an important pro-survival signal to fibroblasts in lung injury [8]. We now demonstrate in αSMA-GFP mice that IGF-1 pathway blockade decreases αSMA + fibroblasts after bleomycin lung injury. In addition to an effect on cell survival, we asked whether IGF-1 induces fibroblast differentiation into myofibroblasts, which may also explain the observed decrease in GFP + and αSMA + cells in our lung injury model with IGF-1 blockade.

Myofibroblasts are specialized fibroblasts that exhibit a contractile phenotype as a result of increased stress fiber formation, αSMA expression, development of mature focal adhesions, and enhanced extracellular matrix deposition [9,23-25]. The phenotypic transformation confers a number of important functional changes that play an important role in lung injury and repair [13,14]. Expression of αSMA, considered a hallmark of the myofibroblast phenotype, contributes to the contractile phenotype that plays an important role in fibrosis [26,27].

Previous studies on the effect of IGF-1 on myofibroblast differentiation and αSMA expression have shown conflicting results. In one study, human fetal lung fibroblasts treated with IGF-1 increased αSMA and collagen I synthesis [11]. In another study, human colonic fibroblast cell lines treated with IGF-1 showed a small increase in αSMA expression that was significantly less than the up-regulation induced by TGF-β1 [12]. Similarly, IGF-1 treatment did not increase αSMA expression in primary human corneal fibroblasts [12,21]. Direct comparison of different studies is complicated by the fact that fibroblasts from different species, organs and stages of development respond differently to fibrogenic stimuli [28-30]. Moreover, recent studies show that extracellular biomechanical properties (i.e. matrix stiffness) regulate myofibroblast differentiation and modulate response to profibrotic cytokines such as TGF-β1 [31-34]. Our data suggest IGF-1 is a profibrotic cytokine under soft extracellular matrix conditions, inducing expression of *Acta2*, *Col1a1*, and *Col3a1*. On stiff substrates, IGF-1 had no effect on αSMA gene or protein expression, alone or synergistically with TGF-β1. On the other hand, the effect of IGF-1 on Col I and III gene and protein expression is maintained in both stiff and soft matrices. We previously demonstrated that IGF-1 stimulation of MLF induced IRS-2 and Akt phosphorylation, suggesting that IRS-2 and PI3 kinase are the major pathways activated by IGF-1 under the conditions tested [8]. In our present study, administration of PI3 kinase inhibitor blocked up-regulation of αSMA, Col I and III in IGF-1-treated MLFs, supporting our previous finding that PI3 kinase is an important downstream pathway in IGF-1-stimulated MLF.

We also investigated whether IGF-1 acted synergistically with TGF-β1, a common profibrotic cytokine. In some studies, TGF-β1 treatment of fibroblasts increased IGF-1 expression [12,21], raising the possibility of an autocrine effect of IGF-1 on αSMA expression. However, in our experiments, co-stimulation with IGF-1 and TGF-β1 did not enhance TGF-β1-mediated αSMA expression, and A12 treatment did not block TGF-β1-induced αSMA expression. Additionally, IGF-1 treatment did not alter TGF-β1 gene expression in MLF. Together, our data suggest the TGF-β1-mediated changes are independent of the IGF-1 pathway.

An interesting finding in our present study is that IGF-1 exerts differential effects on MLF depending on the stiffness of the extracellular matrix. IGF-1 directly up-regulates Col I and III expression in both soft and hard matrices. This finding is consistent with our previously published *in vivo* findings where IGF-1 blockade led to decreased fibrosis as measured by hydroxyproline content at day 21 after bleomycin injury [8]. On the other hand, IGF-1 only regulates αSMA expression in soft matrix conditions. Liu and Tschumerplin previously demonstrated by atomic force microscopy that normal lung parenchyma constitute a soft matrix environment for fibroblasts whereas established fibrotic regions are significantly stiffer [35]. We previously demonstrated a significant increase in IGF-1 mRNA expression during early lung injury in mouse model [8] and increased IGF-1 in bronchoalveolar lavage fluid in early ARDS [7]. We also found that IGF-1 receptor mRNA expression profile is similar to IGF-1 after bleomycin injury (unpublished). Together, these data implicate a temporal influence on the pro-fibrotic function of IGF-1. In early injury, prior to scar formation, IGF-1 may act as a pro-fibrotic cytokine on resident fibroblasts that reside in a compliant extracellular matrix, inducing αSMA expression and collagen deposition by fibroblasts. Later in injury, IGF-1 exerts anti-apoptotic effects on activated myofibroblasts and enhances collagen deposition. Thus, in our *in vivo* model, the decrease in αSMA-GFP + cells observed at day 21 with IGF-1 blockade may be due to decreased myofibroblast differentiation during the early phase of injury and increased apoptosis of fibrogenic myofibroblasts during the resolution phase.

We also found an increase in percentage of fibroblasts exhibiting αSMA-containing stress fibers. These results suggest that IGF-1 promotes the assembly of pre-formed αSMA units into filamentous form (stress fibers), rather than inducing expression of αSMA. Currently, the only demonstrated stimulus for the recruitment of cytosolic αSMA units into stress fibers is mechanical tension mediated by the formation of super-mature focal adhesions [36]. As previously shown, incorporation of αSMA into stress fibers enhances the contractility of myofibroblasts [27], which contributes to the restrictive phenotype seen in fibrotic lung diseases.

Our study has several important limitations. Fibroblasts cultured on stiff substrates such as tissue culture plates invariably become activated, potentially masking a true effect IGF-1 may have on myofibroblast differentiation. Evaluation of the IGF-1 pathway on substrates that mimic the physiologic stiffness of fibrotic lung (~20 kPa Young’s modulus) will be needed to fully assess whether IGF-1 also directly induces the myofibroblast phenotype in stiff lung matrix [31,33,35,37]. Additionally, evaluation of the IGF-1 pathway *in vitro* isolates fibroblasts from their native extracellular matrix and surrounding cellular environment. Cell-matrix interactions and non-fibroblast cellular mediators of myofibroblast differentiation where IGF-1 may also exert its profibrotic effect are absent in our *in vitro* studies. While the presence of collagen I in the growth substrate did not affect MLF responsiveness to IGF-1 treatment in our studies, we cannot exclude the possibility that the observed effects in collagen I gels were due to the three-dimensional substrate versus a two-dimensional substrate.

In summary, IGF-1 blockade decreased myofibroblasts after bleomycin lung injury in αSMA-GFP reporter mice. IGF-1 plays a complex role in lung myofibroblast activation. IGF-1 regulates αSMA expression only in soft substrates while it enhances expression of other myofibroblast markers such as *Col1a1* and *Col3a1* in soft and stiff substrates. We conclude that IGF-1 stimulates myofibroblast differentiation by activating αSMA expression and matrix synthesis. Furthermore, the role of IGF-1 in fibroblast activation is dependent on the biomechanical properties of the extracellular matrix. Our present study highlights the complex biology of fibrosis where the pro-fibrotic effects of different growth factors are dependent on the time course of injury and repair as well as the biomechanical properties of the extracellular matrix.

## Competing interest

LMS holds a patent entitled “Compositions And Methods For The Treatment Of Respiratory Disorders”.

## Authors’ contributions

CH participated in study design, data acquisition and analysis and draft the manuscript, MR participated in data acquisition and analysis, SSL participated in data acquisition and analysis, LMS conceived of the study, and participated in its design and coordination and critically revised drafts of the manuscript. All authors read and approved the final manuscript.

## Supplementary Material

Additional file 1: Figure S1 Effect of A12 on cytokine treatment in stiff substrate. MLF on tissue culture plate were stimulated with IGF-1, TGF-β1 (10 ng/ml), or IGF-1/ TGF-β1 for 24 or 48 hr with or without A12 (40 μg/ml). Negative control is serum free media. Real time PCR analysis of myofibroblast markers *Acta2* and *Col1a1* was performed. Data were normalized to HPRT expression. Y-axis represents fold increase compared to serum-free control (n=3, *p<0.05 compared to serum-free control, **p<0.05 compared to no A12, # not significant compared to no A12). Click here for file

Additional file 2: Figure S2 Treatment with IGF-1 did not affect *Tgfb1* gene expression. MLF on tissue culture plate, soft substrate, or from bleomycin-injured lungs were stimulated with IGF-1, TGF-β1 (10 ng/ml), or IGF-1/TGF-β1 for 24 hr. Negative control is serum free media. Real time PCR analysis of *Tgfb1* was performed. Data were normalized to HPRT expression. Y-axis represents fold increase compared to serum-free control (n=3, mean±SEM). Click here for file
