# Supplementary figures and images for: Role of IGF-1 pathway in lung fibroblast activation
Source: Respir Res. 2013 Oct 8;14(1):102. doi: 10.1186/1465-9921-14-102 (PMC3840605; doi:10.1186/1465-9921-14-102)

# Effect of IGF-1 blockade on stiff substrate

## 24hr

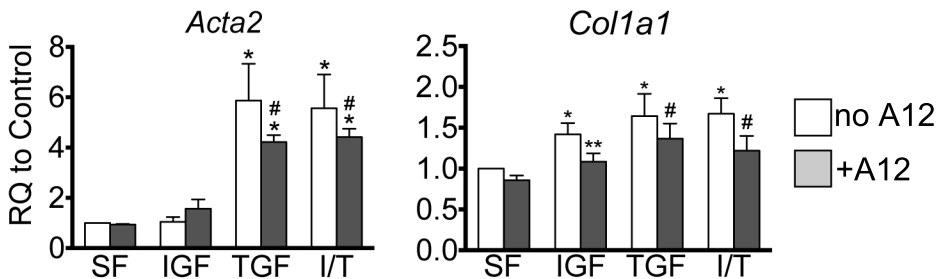

## 48hr

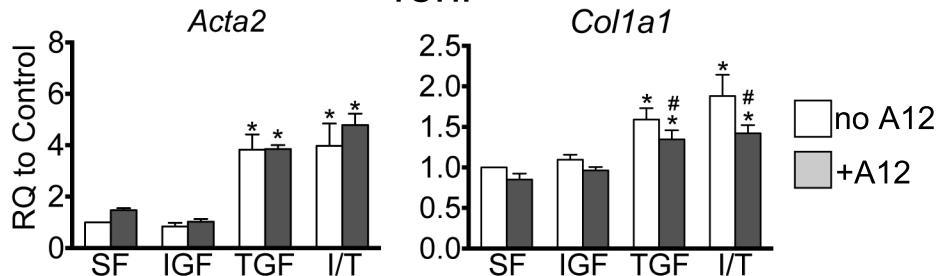

Supplement: Additional file 1: Figure S1 — Effect of A12 on cytokine treatment in stiff substrate. MLF on tissue culture plate were stimulated with IGF-1, TGF-β1 (10 ng/ml), or IGF-1/ TGF-β1 for 24 or 48 hr with or without A12 (40 μg/ml). Negative control is serum free media. Real time PCR analysis of myofibroblast markers Acta2 and Col1a1 was performed. Data were normalized to HPRT expression. Y-axis represents fold increase compared to serum-free control (n=3, *p<0.05 compared to serum-free control, **p<0.05 compared to no A12, # not significant compared to no A12). [file 1465-9921-14-102-S1.pdf]

## Stiff Substrate

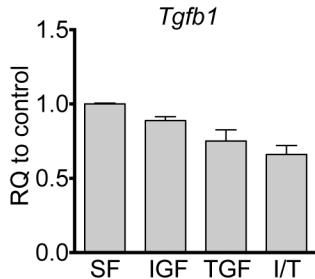

## Soft Substrate

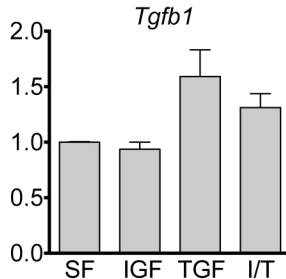

## Bleo Injured

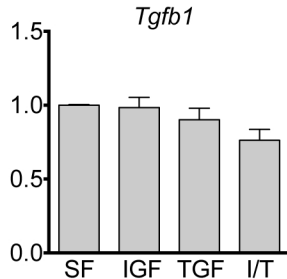

Supplement: Additional file 2: Figure S2 — Treatment with IGF-1 did not affect Tgfb1 gene expression. MLF on tissue culture plate, soft substrate, or from bleomycin-injured lungs were stimulated with IGF-1, TGF-β1 (10 ng/ml), or IGF-1/TGF-β1 for 24 hr. Negative control is serum free media. Real time PCR analysis of Tgfb1 was performed. Data were normalized to HPRT expression. Y-axis represents fold increase compared to serum-free control (n=3, mean±SEM). [file 1465-9921-14-102-S2.pdf]
